# Severe Sepsis Caused by Bacteria That Entered via the Intestinal Tract: A Case of Crohn's Disease in a Child

**DOI:** 10.7759/cureus.9822

**Published:** 2020-08-17

**Authors:** Kasumi Satoh, Manabu Okuyama, Tomoki Furuya, Yasuhito Irie, Hajime Nakae

**Affiliations:** 1 Department of Emergency and Critical Care Medicine, Akita University Graduate School of Medicine, Akita, JPN

**Keywords:** severe sepsis, intensive care unit, child, inflammatory bowel disease, crohn's disease

## Abstract

Typical causes of infection in Crohn's disease (CD) patients include intra-abdominal abscess, microperforation of the intestine, and fistula formation. Use of immunosuppressive drugs and abdominal surgery are often associated with CD sepsis. In this case, an 11-year-old boy who did not receive any concomitant treatment was admitted for evaluation owing to weight loss. On the 22nd day of hospitalization, he suddenly experienced a septic shock and was admitted to the intensive care unit (ICU). *Enterobacter cloacae* was detected in the blood culture. No findings as to the source of the intra-abdominal infection were present. The patient was treated with antibiotics, ventilator management, circulatory management with massive intravenous fluids and vasoactive agents, and blood purification therapy. Suspecting the presence of CD based on weight loss and a history of perianal abscess two years prior, a lower gastrointestinal endoscopy was performed. The results revealed a longitudinal ulcer with skip lesions. His general condition was stabilized, and he was extubated on the seventh day in the ICU. He subsequently began treatment for CD in the general pediatric ward. In conclusion, when a sepsis on account of the intra-abdominal infection is suspected, but the infection focus is not evident and the immunosuppressive background is unclear, CD should be considered. Doing so will ensure that sepsis and CD are treated appropriately.

## Introduction

Crohn's disease (CD) is an inflammatory bowel disease that can affect any part of the gastrointestinal tract from the oral cavity to the anal cavity. Extraintestinal symptoms can occur in areas such as the skin and joints. Infections have also been reported as complications of CD. Typical causes of infection in patients with CD include intra-abdominal abscesses, phlegmons caused by microperforation of the intestine, and formation of fistulae such as perianal and enterovesical fistulae [1,2]. Prior reports have shown that immunosuppressive drug administration and abdominal operation history are important aspects of the patient’s background, as these have been linked to CD infection [3,4]. In this report, we present a case of undiagnosed CD in a child who developed severe sepsis without specific physical or imaging findings, thereby suggesting atypical infection focus.

## Case presentation

An 11-year-old Japanese male (weight, 38.0 kg; height, 149.6 cm) was admitted to the hospital after having lost 3 kg of body weight in one month. He complained of neither subjective symptoms nor abdominal pain. He had undergone surgery for a perianal abscess at the age of nine years, but there were no signs of disease during treatment. He showed elevated serum amylase (AMY) and lipase levels of 524 U/L and 1,114 U/L, respectively. Contrast-enhanced abdominal CT scanning revealed slight pancreatic enlargement. He had a fever once during the course of the disease, but the levels of the pancreatic enzymes spontaneously decreased with fasting. On the 22nd day of hospitalization, he had a fever of 40℃, rigors, and low systolic blood pressure of 70 mmHg. He was, therefore, transported to our hospital for intensive care.

During our examination, he was restless, and his blood pressure, pulse rate, respiratory rate, oxygen saturation, and body temperature were 75/35 mmHg (mean arterial pressure [MAP] 48 mmHg), 150 beats/min, 30 breaths/min, 98% and 38.2°C, respectively. There were no specific physical findings in the chest, abdomen, skin, or anus.

**Figure 1:**
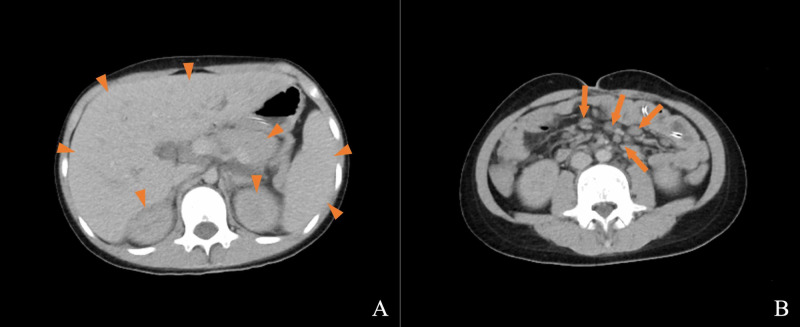
Contrast-enhanced abdominal CT image on the day of the onset of septic shock. Mild swelling was observed in the liver, kidneys, and pancreas (A, arrowhead), and multiple lymph nodes appeared enlarged (B, arrow). There were no obvious infectious findings such as abscesses or intestinal perforations.

Arterial blood gas analysis showed a lactate level of 40 mg/dL, suggestive of lactic acidosis. The leukocyte count was 7,800/μL, the hemoglobin level was 9.2 g/dL, and the platelet count was 98,000/μL. The blood urea nitrogen level was 40.8 mg/dL, the creatinine level was 1.67 mg/dL, and the AMY level was 349 U/L. The lipase, C-reactive protein (CRP), procalcitonin, and blood sugar levels were all abnormal at 372 U/L, 12.99 mg/dL, over 100 ng/dL, and 49 mg/dL, respectively. Coagulation abnormalities were also present, with an activated partial thromboplastin time of 44.4 seconds, a prothrombin time of only 28.2% of the normal range, 44.2 μg/mL of fibrin/fibrinogen degradation products, a D-dimer level of 22.87 μg/mL, a fibrinogen level of 345 mg/dL, and an antithrombin III level of only 61.8% of the normal range. The Sequential Organ Failure Assessment (SOFA) score was 10 points, and the Japanese Association for Acute Medicine disseminated intravascular coagulation (DIC) diagnostic criteria score was 6 points [5,6]. Abdominal CT revealed mild swelling in the liver, kidneys, and pancreas and multiple lymph node enlargements, but no obvious infectious findings such as abscesses or intestinal perforations (Figure 1).

Microbiological testing detected *Enterobacter cloacae* in two sets of blood culture samples and *Enterobacter cloacae*, *Enterococcus faecalis*, and *Proteus mirabilis* in the urine culture sample. Since the urine had a small amount of bacteria and no pyuria was observed, we judged it to be only bacterial carriage.

**Figure 2:**
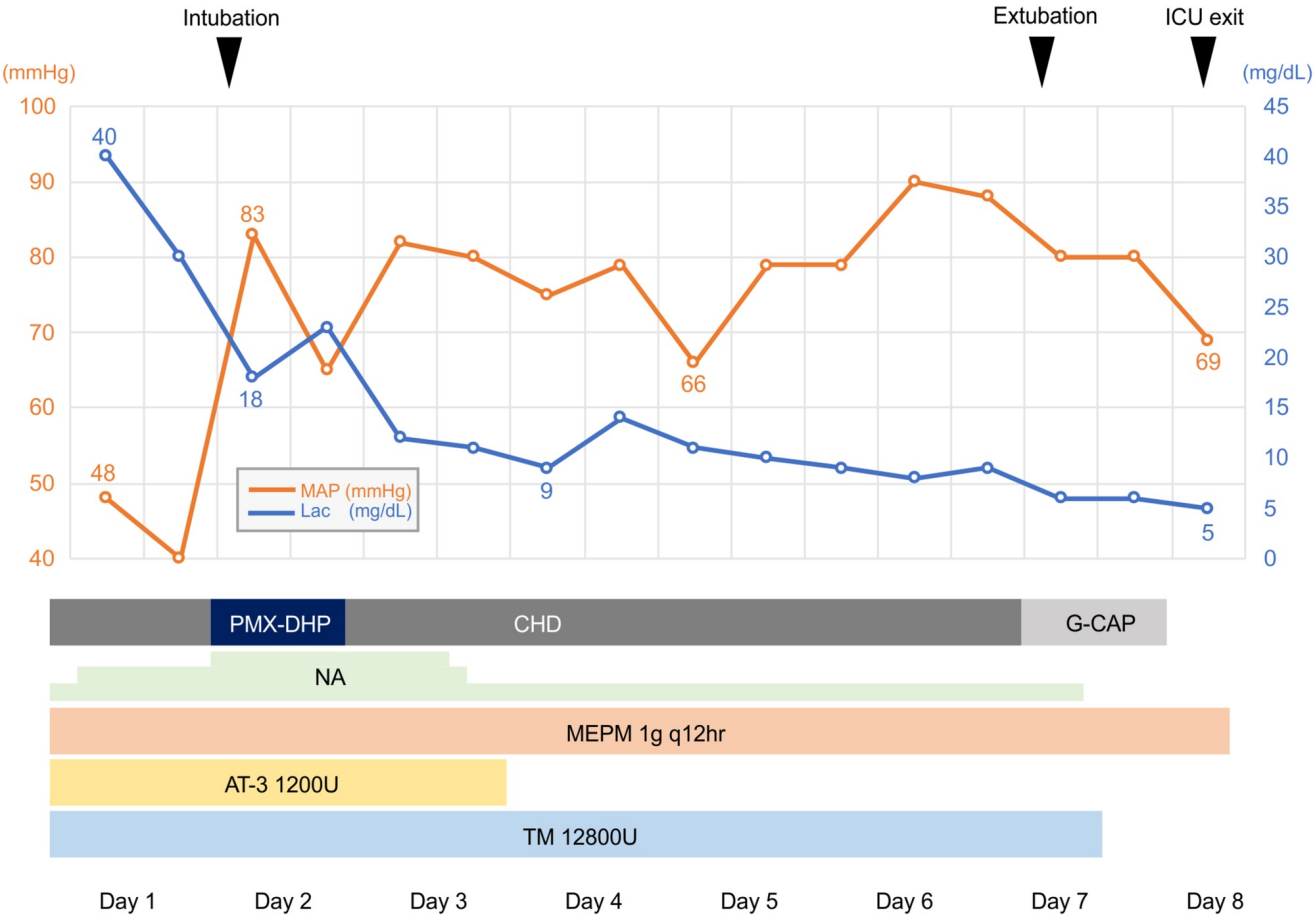
Overview of the course of treatment in the intensive care unit. The number of intensive care unit (ICU) days, treatment, mean arterial blood pressure (MAP) trends, and serum lactate (Lac) level trends are shown. Other abbreviations: MEPM; meropenem, TM; thrombomodulin alfa, NA; noradrenaline, AT; antithrombin, CHD; continuous hemodialysis, PMX-DHP; polymyxin B-immobilized fiber column-direct hemoperfusion, G-CAP; granulocytapheresis.

The patient was admitted to the intensive care unit (ICU) with a diagnosis of septic shock and DIC due to an intra-abdominal infection. Crystalloid fluid infusion, meropenem (MEPM), thrombomodulin alfa (TM), noradrenaline (NA), and antithrombin (AT) were administered (Figure 2). As the patient continued to be anuric despite massive infusions, continuous hemodialysis (CHD) and polymyxin B-immobilized fiber column direct hemoperfusion (PMX-DHP) were introduced. On the second day in the ICU, endotracheal intubation was performed, and he was placed on a ventilator owing to signs of worsening respiratory condition, including tachypnea and nasal alar breathing.

After overnight administration of 4,000 mL of crystalloid fluid and a maximum dose of 0.2 μg/kg/min of NA, a MAP of 65 mmHg was achieved, and serum lactate levels were decreased to 18 mg/dL by day 2 of being in the ICU.

**Figure 3:**
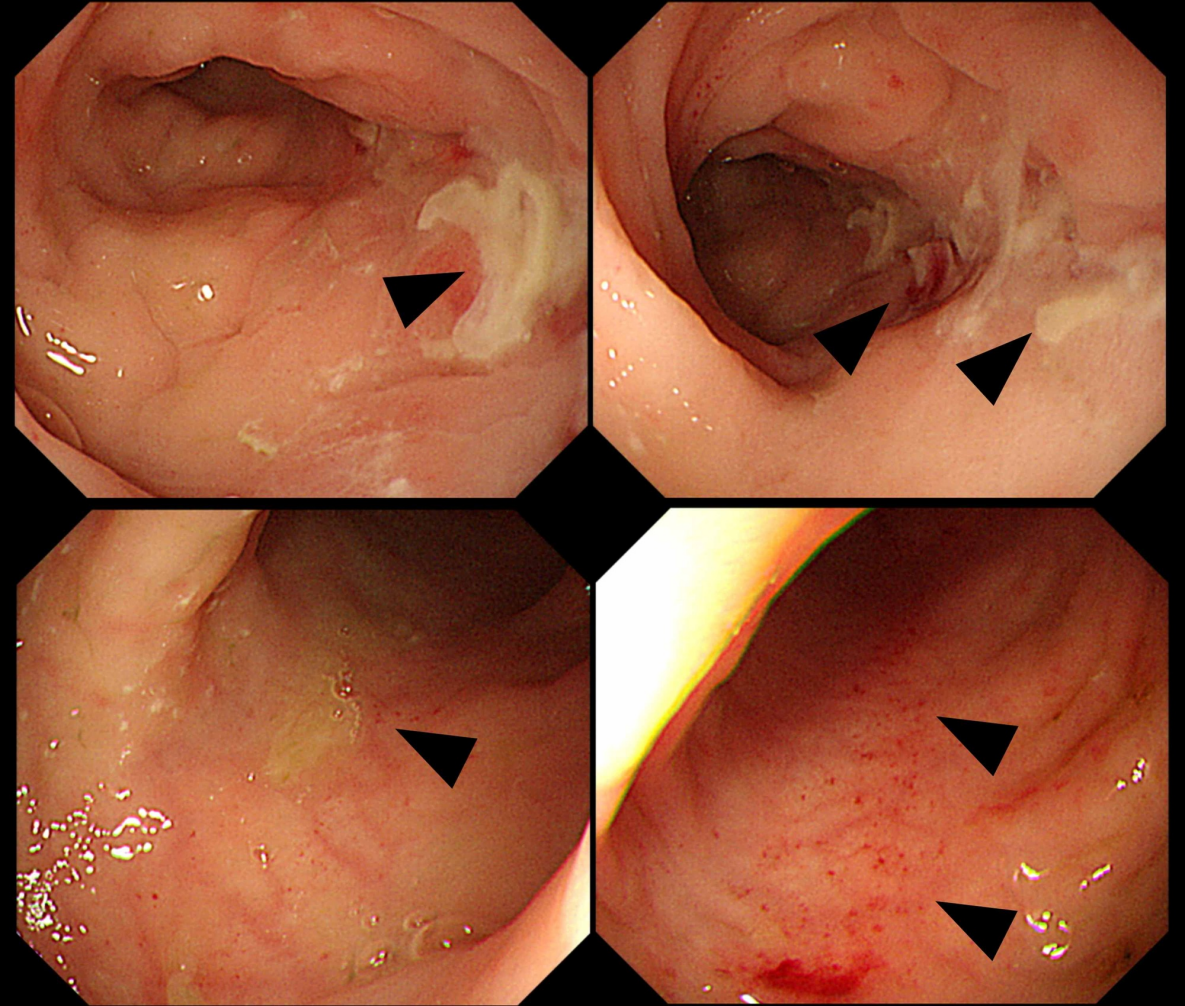
Colonoscopy findings in the intensive care unit. The erosions and ulcers show a tendency to run longitudinally, and skip lesions are present (arrowhead). The imaging findings suggest Crohn's disease.

Colonoscopy was performed to detect the source of the intra-abdominal infection on day 4 of being in the ICU. The erosions and ulcers had a tendency to run longitudinally, and skip lesions were observed (Figure 3). As a result, CD was diagnosed. Since there were no other obvious findings, we assumed that the intestinal tract was the entry point for the infection in the bloodstream.

On day 7 of being in the ICU, the patient was extubated, and the blood purification therapy was terminated after CHD was exchanged for granulocytapheresis (G-CAP).

On the eighth day in the ICU, the patient was transferred to the general pediatric ward for the treatment of CD. On the 117th day of hospitalization, he was discharged in good general condition.

## Discussion

Two important clinical dilemmas were encountered. Despite the absence of the typical infectious findings of CD, bloodstream infection via the intestinal tract can be assumed. CD can cause sepsis in the absence of immunosuppressive drugs or a history of surgery, thereby resulting in a diagnosis of CD.

First, sepsis caused by intestinal infection can occur even if no typical source of CD infection is found. Typical sources of infection in patients with CD are fistulas, abscesses, and phlegmons due to microperforations of the intestine caused by inflammatory processes [1,2]. The key point, in this case, is that the fatal, bacterial bloodstream infection in the intestine had a cause, despite the lack of any of those typical findings. Exclusionary diagnostic considerations led us to assume that the bacteria entered through the injured intestinal mucosa. To the best of our knowledge, no reports similar to the present case exist. In light of the above explanation, we suggest that CD be considered in sepsis of undetermined cause due to bloodstream infection with intestinal bacteria.

Second, CD can cause severe sepsis irrespective of immunosuppressive drug administration or surgery. However, the development of sepsis can also lead to a diagnosis of CD, as in this case. As the patient history relates to infection with CD, the cause is often treatment-related, such as abdominal surgery history and prior treatment with immunosuppressive drugs [3,4]. This case provides valuable insights in the sense that severe sepsis occurred in a case of CD that had not been treated or even diagnosed. In this case, owing to his weight loss and history of perianal abscesses, we suspected that CD played a role in his *Enterobacter cloacae* sepsis. We were able to perform a gastrointestinal endoscopy in the ICU, which led to the diagnosis of CD. He did not present with the common complaints of diarrhea, bloody stools, or fever; therefore, his CD went undiagnosed for a relatively long period of time. It is difficult to diagnose CD, especially in pediatric patients, as they often present with non-classical symptoms such as stunted growth and extraintestinal symptoms [7]. When a sepsis patient presents without a clear cause of infection, it is pertinent to consider CD. This is especially true in children.

## Conclusions

CD can be associated with patients who are suspected of having intra-abdominal infections and have no obvious clinical findings. If severe sepsis occurs in a patient without a history of treatment with immunosuppressive drugs, CD should be considered. These two important points have valuable clinical implications in the treatment of sepsis as well as the detection of undiagnosed CD.
